# Computational investigation of DNA packing in confinement

**DOI:** 10.1186/1471-2105-13-S12-A22

**Published:** 2012-07-31

**Authors:** Uta Ziegler, Yuanan Diao, Claus Ernst, Anthony Montemayor

**Affiliations:** 1 Department of Mathematics and Computer Science, Western Kentucky University, Bowling Green, KY 42101, USA; 2 Department of Mathematics and Statistics, University of North Carolina, Charlotte, Charlotte, NC 28223, USA

## Background

High-density packing of DNA in nature, for example in mutant P4 bacteriophage viruses and the resulting entanglement (= knots) of the packaged DNA has been a focus of recent research across several disciplines [1]. Published data allows the conclusion that the ends of the DNA packaged in P4 meet in the capsid creating a circular molecule and that a high percentage of knots is formed due to the effect of the capsid's confinement. This report describes a computational investigation of packing DNA in confinement.

## Materials and methods

In this study DNA is modeled as a freely-jointed closed chain of unit-length segments, called a polygon and confinement is modeled by a sphere. Packaging is modeled by randomly generating polygons which fit inside the sphere. In a 'local' model, obeying confinement only matters when the segment might actually breach it [2]. A 'global' model biases the generation of every segment to avoid a future breach of the boundary [3]. For each model its (cumulative) probability density function - in terms of the distance *d* of the next vertex from the origin - was derived which is the basis for the algorithm generating such polygons with uniform probability.

### The algorithm

The starting and the end vertex of the polygon is the origin and the second vertex is on the unit sphere. For each successive vertex, use the cumulative probability density function to select uniformly the distance d of the next vertex from the origin. The next vertex is then chosen uniformly on the intersection circle of the unit sphere around the current vertex and the sphere with radius d around the origin. The second to last vertex is chosen uniformly from the intersection circle of the unit spheres around the current vertex and the origin.

## Results

**Figure 1:**
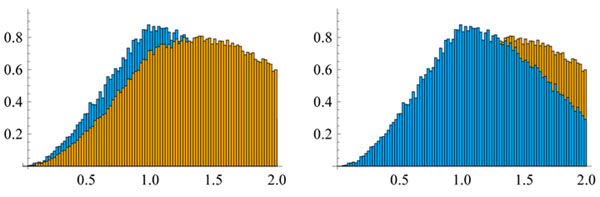
The histograms (normalized to unit area) of the distributions of the distance of the vertices to the origin for the global (darker color) and local method.

**Figure 2:**
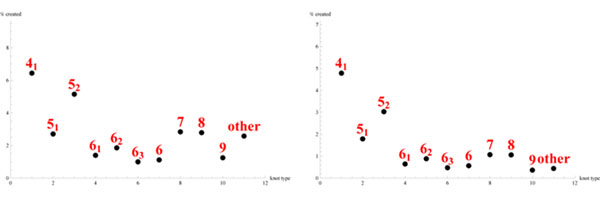
The knots entangled in the polygons for the local (right) and global (left) methods.

10000 polygons were generated with various lengths and in confinement with radii from 1 to 4.5 in 0.5 increments. The two different models lead to significant differences with respect to the distribution of the polygons in the sphere and with respect to the entanglement. To measure the former histograms of the distribution of the vertices were computed; for a typical result see Figure 1. For the latter, the knots formed by the polygons were extracted (Figure 2). About 48.8% of the polygons were knotted in the global method compared with 46.4% in the local method (20 segment polygons in a sphere of radius 1).

## References

[B1] Arsuaga J Vazquez M Trigueros S Sumners DW Roca J Knotting probability of DNA molecules confined in restricted volumes: DNA knotting in phage capsids Proc Natl Acad Sci U S A 2002 99 5373 5377 10.1073/pnas.032095099 11959991 PMC122776

[B2] Diao Y Ernst C Montemayor A Ziegler U Generating equilateral random polygons in confinement J. Phys A: Math Theor 2011 44 40 405202 10.1088/1751-8113/44/40/405202

[B3] Diao Y Ernst C Montemayor A Ziegler U Generating equilateral random polygons in confinement II preprint

